# The Dutch Obesity Intervention in Teenagers (DOiT) cluster controlled implementation trial: intervention effects and mediators and moderators of adiposity and energy balance-related behaviours

**DOI:** 10.1186/s12966-014-0158-0

**Published:** 2014-12-24

**Authors:** Femke van Nassau, Amika S Singh, Ester Cerin, Jo Salmon, Willem van Mechelen, Johannes Brug, Mai JM Chinapaw

**Affiliations:** Department of Public & Occupational Health and EMGO Institute for Health and Care Research, VU University Medical Center, Amsterdam, The Netherlands; Centre for Physical Activity and Nutrition Research, Deakin University, Burwood, Victoria Australia; Department of Epidemiology and Biostatistics, EMGO Institute for Health and Care Research, VU University Medical Center, Amsterdam, The Netherlands

**Keywords:** Adolescents, Mediation, Intervention, Prevention, Energy balance, Implementation, Dissemination

## Abstract

**Background:**

The Dutch Obesity Intervention in Teenagers (DOiT) programme is an evidence-based obesity prevention programme tailored to adolescents attending the first two years of prevocational education in the Netherlands. The initial programme showed promising results during an effectiveness trial. The programme was adapted and prepared for nationwide dissemination. To gain more insight into the process of translating evidence-based approaches into ‘real world’ (i.e., ‘natural’) conditions, our research aims were to evaluate the impact of the DOiT-implementation programme on adolescents’ adiposity and energy balance-related behaviours during natural dissemination and to explore the mediating and moderating factors underlying the DOiT intervention effects.

**Methods:**

We conducted a cluster-controlled implementation trial with 20 voluntary intervention schools (n=1002 adolescents) and 9 comparable control schools (n = 484 adolescents). We measured adolescents’ body height and weight, skinfold thicknesses, and waist circumference. We assessed adolescents’ dietary and physical activity behaviours by means of self-report. Data were collected at baseline and at 20-months follow-up. We used multivariable multilevel linear or logistic regression analyses to evaluate the intervention effects and to test the hypothesised behavioural mediating factors. We checked for potential effect modification by gender, ethnicity and education level.

**Results:**

We found no significant intervention effects on any of the adiposity measures or behavioural outcomes. Furthermore, we found no mediating effects by any of the hypothesised behavioural mediators. Stratified analyses for gender showed that the intervention was effective in reducing sugar-containing beverage consumption in girls (B = -188.2 ml/day; 95% CI = -344.0; -32.3). In boys, we found a significant positive intervention effect on breakfast frequency (B = 0.29 days/week; 95% CI = 0.01; 0.58). Stratified analyses for education level showed an adverse intervention effect (B = 0.09; 95% CI = 0.02; 0.16) on BMI z-scores for adolescents attending the vocational education track.

**Conclusions:**

Although not successful in changing adolescents’ adiposity, the DOiT-implementation programme had some beneficial effects on specific obesity-related behaviours in subgroups. This study underlines the difficulty of translating intervention effectiveness in controlled settings to real world contexts. Adaptations to the implementation strategy are needed in order to promote implementation as intended by the teachers.

**Trial registration:**

Current Controlled Trials ISRCTN92755979.

**Electronic supplementary material:**

The online version of this article (doi:10.1186/s12966-014-0158-0) contains supplementary material, which is available to authorized users.

## Background

In many countries, interventions are needed for curbing the prevalence of overweight and obesity in youth [1-4]. Overweight and obesity occur due to a prolonged positive energy balance, i.e., when energy intake exceeds energy expenditure [5]. Because childhood overweight and obesity are associated with many health risks during childhood and adulthood, and since they track into adulthood [6-8], prevention of overweight in youth is a major public health priority.

Schools are regarded as a convenient and practical setting for implementing interventions as they allow access to almost all children and adolescents regardless of ethnic and socioeconomic background [9]. Evidence-based multi-component school-based interventions that target multiple obesity-related behaviours, and that combine educational and environmental approaches seem to be most promising [9,10]. However, only a small number of such interventions have been delivered and evaluated during natural dissemination; that is when interventions are disseminated without strong support from a research team and, therefore, follow the natural course of adoption, implementation and maintenance [11,12]. Insight into effectiveness, the working mechanisms and subgroup effectiveness of interventions when they are introduced under less controlled and directed conditions is crucial in order to more effectively translate evidence-based interventions into practice [13].

The evidence-based Dutch Obesity Intervention in Teenagers (DOiT) programme is an example of a multi-component intervention that has been disseminated in a natural way. DOiT is an ongoing school-based obesity prevention programme for adolescents attending the first two years of prevocational education [14,15]. Prevocational education is secondary education for adolescents aged 12–16 with four tracks: theoretical programme, combined programme, middle-management vocational programme, basic vocational programme [16]. The DOiT programme targets both sides of the energy-balance equation (energy intake and energy expenditure) in order to prevent overweight and obesity in youth [14]. Based on self-regulation theory [17], adolescents’ energy balance-related behaviours (EBRBs) are targeted in order to maintain or achieve a healthy weight.

In a randomised controlled trial with follow-up measurements at 8, 12 and 20 months conducted in 2003–2005, the DOiT-effectiveness programme showed promising results on some measures of adiposity (reduced skinfold thickness in boys and girls, and reduced waist circumference (WC) in boys) and EBRBs (a reduction in sugar-containing beverage (SCB) consumption in both boys and girls, and a reduction in screen time in boys) [18,19]. Mediation analyses showed that SCB consumption mediated the intervention effects on BMI [20].

In 2009–2010, the DOiT-effectiveness programme was adapted and prepared for nationwide dissemination [21]. As the initial DOiT-effectiveness programme was shown to be effective [18,19], all of its content, core elements and practical strategies, e.g., goal setting, modelling and feedback, were retained in the adapted version [14]. The effectiveness of the adapted DOiT-implementation programme during natural dissemination has not been investigated yet. Consequently, our research aims were: 1) to evaluate the intervention effects of the DOiT-implementation programme on adolescents’ adiposity and EBRBs during natural dissemination; 2) to test the EBRB mediating factors underlying the DOiT-implementation intervention effects on adolescents’ adiposity; and 3) to explore whether gender, ethnicity and adolescents’ education level moderated the mediated effects of the intervention.

## Methods

This evaluation is an integral part of the ongoing natural dissemination process of the evidence-based DOiT-implementation programme throughout the Netherlands. The current study included data from a 20-month cluster-controlled trial. Details of the aims, design and methods have been published elsewhere [15]. Data collection involved adolescents’ measures of adiposity and questionnaires completed during class-time. The Medical Ethical Committee of the VU University Medical Center approved the study protocol in which we applied a passive consent procedure for adolescents.

### Procedures and participants

Since August 2011, the DOiT-implementation programme has been made available to all schools in the Netherlands as a voluntary add-on to the mandatory curriculum. In order to recruit Dutch prevocational schools, a DOiT support office was installed. The DOiT support office employee actively recruited schools by activities such as posting news items on relevant websites, or in digital mailings and by being present at local meetings of relevant stakeholders. Additionally, the DOiT support office employee informed prevocational schools by sending a DOiT introductory package [21]. After a school had purchased the DOiT materials (i.e. 7 euro per adolescent for the two-year programme), the school was invited to participate in the present study, until a sample of 20 volunteering schools was reached. Because of the nature of the program roll-out, the number of schools that were approached is unknown.

In order to recruit control schools, we asked all intervention schools to provide the name of comparable schools in their area not involved in the programme. A total of nine control schools were recruited. At the intervention and control schools, we invited all adolescents in three classes nominated by the school to participate in the evaluation study; no exclusion criteria were set for participants.

Sample size calculations were based on anticipated changes in BMI; 510 adolescents per group were needed to detect a relevant difference in BMI (0.25 kg/m^2^) between the intervention and control groups. We increased the sample size (+25%) to account for clustering effects derived from a cluster-controlled design and for dropouts. The number of intervention schools was greater than control schools to increase the power of the accompanying process evaluation study [15].

### Intervention

DOiT is a school-based obesity prevention programme for 12 to 14-year olds, developed according to the Intervention Mapping protocol [14,22]. Based on the conducted process evaluation in 2003–2005 and additional interviews with teachers, adolescents and parents in 2009, we made adapations to the DOiT-effectiveness programme. Adaptations to the programme among others were an extension of the programme from one to two school years, adding daily breakfast consumption as an additional potential target behaviour, and adding a parental component to the programme. Further, we developed a 5-minute instruction video to guide teachers through the programme and materials. Two different versions of the DOiT-implementation programme were developed; tailored to the two higher (theoretical) and two lower (vocational) subtracks of the prevocational education system in the Netherlands [21].

The aim of the DOiT programme was to increase awareness and to induce behavioural changes concerning EBRBs in order to prevent overweight and obesity in adolescents. The DOiT-implementation programme focused on five EBRBs: (1) reducing intake of SCB; (2) reducing intake of high-energy snacks/sweets; (3) reducing screen time; (4) increasing levels of physical activity (i.e., active transport to school and sports participation) and (5) consuming a daily breakfast [14].

The DOiT-implementation programme consisted of 12 fixed theory lessons and four physical education lessons (i.e., 16 lessons equally divided over two school years) and three optional lessons. The lessons in the first year aimed at increasing awareness and knowledge of EBRBs. The lessons in the second year focused on increasing awareness and acting upon the influence of the (obesogenic) environment. The parental component focused on increasing social support of the parents and on raising awareness of the availability and accessibility of healthy products and activities in the home environment. We developed materials and a few programme activities to improve parental engagement: an information booklet, homework assignments that adolescents needed to conduct with their parents, information for parents on the DOiT website and an optional parents’ meeting.

The DOiT class room materials included a ‘schoolbook’ accompanied by worksheets and a student toolkit (pedometer, food/exercise diary and an online computer-tailored advice).

To facilitate the implementation process, we provided a 7-step implementation strategy with accompanying materials to teachers on the DOiT website. Furthermore, DOiT was supported by an extensive teacher manual with a login for extra materials provided at the DOiT website. The development and content of the DOiT programme have been described in more detail elsewhere [14,21]. Control schools were asked to maintain their regular curriculum.

### Measures

Baseline data collection took place between September and November 2011 (T0) and follow-up measurements were performed between April and June 2013 (T1). We examined measures of adiposity according to a standardised protocol; the examination consisted of measurements of body weight and height, WC, and skinfold thicknesses. Hypothesised mediators of intervention effects (dietary and physical activity behaviours) were assessed by questionnaire. Before the survey administration, a researcher explained the procedures. Adolescents needed approximately one school lesson (i.e., 45 minutes) to complete the questionnaire. To minimise seasonal influences, data were collected from intervention and control schools concurrently and all measurements were performed within a 10-week period. For practical reasons, the research team was not blinded to the group assignment.

#### Demographic data

Gender, date of birth, ethnicity and level of prevocational education were assessed by adolescent self-report. Ethnicity was categorised into Western or Non-Western based on the country of birth of the parents, according to the Dutch Central Bureau for Statistics method. If at least one of the parents was born outside Europe (excluding Turkey), North-America, Oceania, Indonesia or Japan, ethnicity was categorised as Non-Western [23]. We dichotomised adolescents’ education level into the vocational (i.e., middle-management and basic vocational programme) and the theoretical (i.e., theoretical and combined programme) track, according to the four subtracks of prevocational education in the Netherlands [16].

#### Adiposity measurements

We measured body weight and height, skinfold thickness (i.e., triceps, biceps, suprailiac, and subscapular), and WC. Before the assessment, a researcher explained the procedures. Adolescents were measured wearing underwear only. If adolescents did wear outer clothes (n = 63 (T0) and n = 124 (T1)), we adjusted body weight for clothing according to standardised clothing mass values (i.e., jeans/pants = +0.75 kg; shorts = +0.30 kg; sweater = +0.30 kg; t-shirt = +0.20 kg). Body weight was measured and recorded with a calibrated electronic flat scale (Seca 861, Hamburg, Germany), levelled after each placement, with an accuracy of 0.1 kg. Body height was measured with an accuracy of 1 mm with a portable stadiometer (Leicester Height Measure). We calculated BMI as weight in kilograms divided by the square of height in meters (kg/m^2^). We used the BMI cut-off values for weight status (underweight, normal weight, overweight, and obesity) based on the International Obesity Task Force criteria (IOTF) [24]. BMI was also converted into sex- and age-specific BMI z-scores [25].

Skinfold thickness was measured to the nearest 0.2 mm using a Harpenden Skinfold Caliper [26]. All measurements were done on the left side of the adolescent. Measurements were taken twice and repeated if the first two measurements differed by more than 1.0 mm. Each skinfold thickness was calculated as the average of the two nearest measurements. Next, the sum of four skinfolds was calculated. We measured WC with a Seca 201 measure (Seca, Hamburg, Germany) with an accuracy of 0.1 cm. Before each measurement period, we determined the intrarater and interrater reliability of the skinfold thickness and WC measurements. Values for intrarater reliability varied from 0.90 to 0.99 (skinfold thickness) and 0.97 to 0.99 (WC). Values for interrater reliability varied from 0.85 to 0.99 (skinfold thickness) and 0.96 to 0.99 (WC).

#### EBRBs questionnaire

Six behavioural mediators were assessed in the DOiT questionnaire: consumption of SCB, consumption of high-energy snacks/sweets, sport participation, active transport to school, screen time (TV viewing and computer use) and breakfast consumption. Details on the questionnaire can be found elsewhere [27]. The questionnaire showed good test-retest reliability and moderate to good construct validity for the majority of items. Since the items on physical activity (‘sports participation after school time’) of the original DOiT questionnaire showed only moderate test-retest reliability, we used the questions of the QAPACE questionnaire to assess sport participation in the present study [28]. Adolescents indicated which sport they usually participated in during after-school hours (both in a club setting and during leisure-time), how many times per week they participated, and how many hours they dedicated to the sport each time. Frequency and quantity of the reported behaviours were multiplied to obtain estimates of mean daily behaviour.

### Statistical analyses

Descriptive statistics (mean, standard deviations and proportions) were computed to present the baseline characteristics of the participants. Due to the skewed distributions of data, we also calculated the median and interquartile range (IQR) for the baseline values. The analytical sample included adolescents with data from the questionnaire and at least one complete measure of adiposity, that was assessed at both time points (n = 1486). We thus conducted a complete cases analysis. An intention to treat analysis would require imputing missing values at post-test using baseline data (i.e. assuming no change between baseline and follow-up). Such imputation for missing data for adolescents who are still growing –and for whom changes are thus to be expected with or without an intervention- would not lead to valid estimates of missing values and may thus bias the results of the present study [29].

Multilevel linear and logistic regression analyses were conducted to test for differences between the intervention and control group in demographics and measures of adiposity. We used Mann–Whitney tests to test for differences in EBRBs at baseline, due to the skewed distributions of data. Differences between those who were included for analyses and those who were not included due to dropping out from the study or incomplete data were tested using similar analyses. Self-reported values for SCB consumption (n = 123 at T0; n = 186 at T1), screen time (n = 80 at T0; n = 46 at T1) and sports participation (n = 52 at T0; n = 110 at T1) exceeding the 95^th^ percentile of the respective sample distributions were treated as extreme outliers and replaced by the values representing the 95^th^ percentile of the distribution.

#### Intervention effects on measures of adiposity and EBRBs

We performed a series of multilevel linear and logistic regression analyses to estimate the intervention effect on adolescents’ adiposity and EBRBs. We defined three levels in our multi-level analyses: 1) adolescent; 2) class; and 3) school. This analysis technique enables regression coefficients to be adjusted for clustering of observations within one school and class. Analyses were adjusted for baseline values, gender, age, ethnicity and education level. For all analyses a two-tailed significance level of 0.05 was used. Multilevel regression models were performed using MLwiN 2.22.

#### EBRBs as mediators of intervention effects on adiposity

The working mechanism of DOiT was assessed by the product-of-coefficient method [30]. Associations between each of the potential mediators (i.e., EBRBs) (a-path) and each of the measures of adiposity (b-path) were examined, adjusted for intervention condition (c’-path). The mediated effect of each mediator was computed using the product-of-coefficients method by multiplying coefficients for the a- and b-paths (a*b). The statistical significance of the mediated effect was estimated by dividing the product-of-coefficient by its standard error using Sobel’s standard error (SE) formula (√ a^2^ * SEb^2^ + b^2^ * SEa^2^) [31]. The percentage of the intervention effect mediated by a specific mediator was computed by dividing the product of the coefficients for the a-and b-path by the total intervention effect ((a*b)/c-path). Since mediating effects can still exist without a significant intervention effect on the outcome [32], mediation analyses were also conducted in absence of a significant main effect. Further, we built a multiple mediator model, including all mediators into the same model.

#### Moderating effects of gender, ethnicity and education level

We checked potential effect modification by gender, ethnicity and adolescents’ education level by including group X gender, group X ethnicity, and group X education level interaction terms in the regression analyses, respectively. Stratified analyses were conducted when appropriate.

## Results

**Figure 1:**
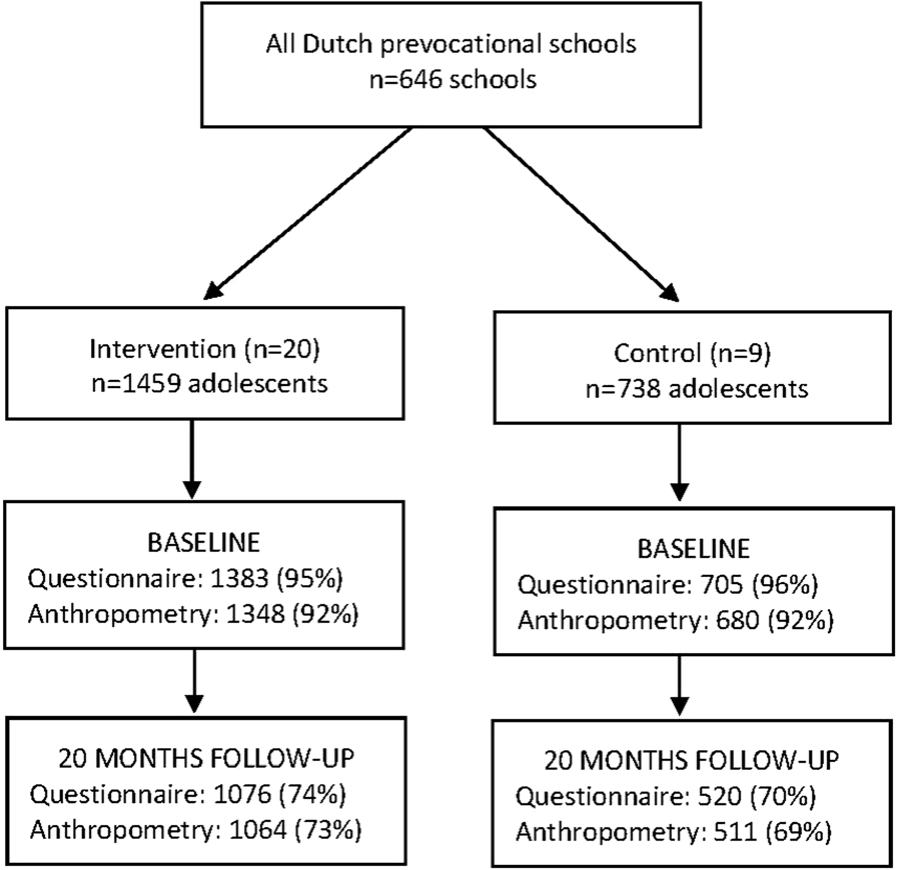
**Flow diagram of recruitment, and participation of adolescents in the DOiT implementation study.**

At baseline, 2088 adolescents completed the questionnaire, of whom 2028 also completed measures of adiposity. At 20-months follow-up, 1596 completed the questionnaire and 1575 also completed measures of adiposity (see Figure 1). In total, 1486 adolescents (n = 484 in the control group; n = 1002 in the intervention group) were included in the analyses (questionnaire data and at least one measure of adiposity provided at both time points).

Reasons for drop-out included: (1) absence from school during the lesson when the measurement was taken; (2) moved to a different school or class; or (3) refused to participate at follow-up. Due to student turnover at follow-up, we could not trace the exact number of drop-outs or their reason for dropping out. Further, one school (n = 61 adolescents) declined to participate at follow-up, due to lack of support from the management to continue participation in the study. Compared to participants who had dropped out, adolescents who had completed measurements on both occasions were significantly younger (12.9 vs. 13.2 years), had significantly lower BMI values (19.5 vs. 20.1 kg/m^2^) and smaller WC (70.9 vs. 72.0 cm), and had reported significantly more active transport per day (mean 37.7 vs. 34.7 min/day). There was no significant difference between the number of drop-outs between the intervention and control groups.

In the intervention group, age (12.8 vs. 13.0 years) and the proportion of Western adolescents were significantly lower (70% vs. 82%), and the portion of adolescents attending the vocational subtrack was significantly higher (51% vs. 44%) compared to the control group at baseline.

In Table 1, the baseline and follow-up values for measures of adiposity and EBRBs are presented. Boys in the intervention group reported significantly fewer minutes of active transport compared to boys in the control group. Girls in the intervention group reported significantly lower consumption of SCB compared to girls in the control group. No significant differences for the other variables between the intervention and control group were found.

**Table 1:** **Baseline and 20 month follow-up measures of adiposity and EBRBs in the intervention and control group**

	**Baseline**										**20 month follow up**									
	**Intervention**					**Control**					**Intervention**					**Control**				
**Weight status (IOTF)** ^**b**^																				
Underweight (%)																				
*Boys*	7.6					8.9					7.4					9.7				
*Girls*	5.9					10.3					6.5					9.1				
Normal weight (%)	71.8					68.3					73.9					71.7				
*Boys*	71.9					67.4					72.8					67.8				
*Girls*	71.8					69.1					74.9					75.5				
Overweight (%)	18.1					18.4					15.4					16.6				
*Boys*	17.1					18.6					15.8					19.1				
*Girls*	19.1					18.1					15.0					14.1				
Obese (%)	3.3					3.8					3.8					2.3				
*Boys*	3.4					5.1					4.0					3.4				
*Girls*	3.2					2.5					3.6					1.2				
**Measures of adiposity**																				
	**n**	**Mean**	**SD**	**Median**	**IQR**	**n**	**Mean**	**SD**	**Median**	**IQR**	**n**	**Mean**	**SD**	**Median**	**IQR**	**n**	**Mean**	**SD**	**Median**	**IQR**
BMI (kg/m^2^)	998	19.5	3.4	18.7	4.3	479	19.6	3.3	19.1	4.3	996	20.5	3.5	20.0	4.2	477	20.4	3.3	20.0	4.3
*Boys*	474	19.1	3.5	18.1	4.1	236	19.6	3.4	19.0	4.3	475	20.1	3.6	19.5	4.0	236	20.4	3.4	19.9	4.5
*Girls*	524	19.8	3.4	19.2	4.0	243	19.5	3.2	19.1	4.4	521	20.9	3.5	20.4	4.0	241	20.4	3.1	20.1	4.0
BMI z-score WHO^a^	998	0.2	1.2	0.1	1.6	479	0.2	1.2	0.3	1.6	998	0.2	1.2	0.1	1.6	479	0.2	1.2	0.3	1.6
*Boys*	474	0.1	1.2	0.0	1.7	236	0.3	1.2	0.4	1.8	474	0.1	1.2	0.0	1.7	236	0.3	1.2	0.4	1.8
*Girls*	524	0.2	1.1	0.2	1.5	243	0.1	1.1	0.0	1.5	524	0.2	1.1	0.2	1.5	243	0.1	1.1	0.0	1.5
Waist circumference (cm)	1001	70.7	9.5	68.5	10.7	482	71.3	9.7	69.4	11.3	999	73.1	9.4	71.0	10.2	482	73.0	9.3	71.5	10.9
*Boys*	477	71.2	10.2	68.2	12.0	236	72.7	10.3	70.5	12.3	476	74.1	10.0	71.8	11.9	237	74.9	10.2	73.0	11.8
*Girls*	524	70.2	8.7	68.9	10.3	246	70.0	8.9	68.1	10.0	523	72.2	8.7	70.5	9.7	245	71.1	7.9	70.0	10.2
Sum skinfolds (mm)	1000	46.4	23.9	39.0	27.4	484	46.4	25.6	39.2	27.0	998	51.2	27.7	44.4	31.0	478	50.1	26.4	43.3	30.9
*Boys*	475	43.1	25.3	33.4	27.9	237	43.4	25.8	33.9	27.2	477	42.5	26.5	32.4	23.1	237	43.6	28.0	33.9	25.3
*Girls*	525	49.3	22.2	44.1	25.1	247	49.3	25.2	43.0	26.3	521	59.2	26.4	52.9	27.7	241	56.5	23.1	52.1	27.7
**Measures of EBRBs**																				
SCB consumption (ml/day)	911	1219.0	865.1	1011.4	1145.7	453	1304.6	872.9	1085.7	1164.3	866	1126.3	877.0	900.0	1074.3	435	1195.7	865.8	942.9	1121.4
*Boys*	428	1456.4	1236.4	1164.6	1255.0	220	1501.7	1152.2	1232.9	1326.4	407	1464.2	1339.9	1087.1	1192.9	208	1338.0	1112.4	1087.9	1109.1
*Girls*	**483**	**1105.5**	**823.1**	**912.9**	**971.4**	**233**	**1291.5**	**1121.1**	**942.9**	**935.7**	**459**	**974.7**	**903.4**	**742.9**	**978.6**	**227**	**1259.1**	**1299.0**	**857.1**	**1114.3**
High energy snacks/sweets (portion/day)	915	2.1	2.0	1.4	2.0	453	2.2	2.1	1.5	2.2	902	1.9	2.1	1.3	1.9	459	1.8	1.7	1.2	1.9
*Boys*	434	2.2	2.2	1.5	2.3	221	2.3	2.3	1.4	2.4	419	2.0	2.0	1.4	2.1	221	1.9	1.8	1.3	1.9
*Girls*	481	1.9	1.9	1.3	1.6	232	2.2	1.8	1.6	2.2	483	1.8	2.1	1.2	1.6	238	1.7	1.7	1.2	1.9
Breakfast consumption (days/week)	991	5.8	1.9	7.0	2.0	480	5.7	2.0	7.0	2.0	994	5.8	1.9	7.0	2.0	484	5.7	2.1	7.0	2.0
*Boys*	471	5.7	1.8	7.0	2.0	236	5.7	1.9	7.0	2.0	**471**	**6.0**	**1.8**	**7.0**	**1.0**	**237**	**5.7**	**2.1**	**7.0**	**2.0**
*Girls*	520	5.8	1.9	7.0	2.0	244	5.7	2.0	7.0	2.0	523	5.7	2.1	7.0	2.0	247	5.7	2.1	7.0	2.0
Screen time behaviour (min/day)	940	250.8	129.5	231.4	180.0	467	259.9	133.3	248.6	205.7	966	236.3	129.9	214.3	180.0	475	228.1	137.4	197.1	188.6
*Boys*	453	271.6	143.7	257.1	184.3	227	278.0	149.1	274.3	201.4	457	273.6	141.7	257.1	180.0	232	256.7	157.0	231.4	214.3
*Girls*	487	239.3	134.6	214.3	180.0	240	251.0	136.1	214.3	197.1	509	210.3	131.5	188.6	167.1	243	209.9	137.1	175.7	167.1
Active transport to school (min/day)	**985**	**36.4**	**29.6**	**31.0**	**40.0**	**480**	**40.3**	**29.4**	**31.0**	**40.0**	977	35.4	29.1	31.0	40.0	480	40.8	31.4	31.0	40.0
*Boys*	**467**	**38.2**	**31.5**	**31.0**	**40.0**	**235**	**45.9**	**32.7**	**31.0**	**55.0**	464	36.3	29.6	31.0	40.0	236	47.3	34.8	31.0	60.0
*Girls*	518	34.8	27.6	31.0	40.0	245	35.0	24.8	31.0	40.0	513	34.5	28.7	31.0	40.0	244	34.5	26.2	31.0	40.0
Sports participation (min/day)	967	49.0	45.9	38.6	55.7	468	48.7	46.5	38.6	54.6	920	49.7	46.0	38.6	64.3	457	51.3	52.3	38.6	68.6
*Boys*	461	61.9	55.5	51.4	64.3	229	57.0	57.8	38.6	62.1	434	63.3	58.1	51.4	64.3	228	65.5	79.3	38.6	76.1
*Girls*	506	41.1	47.9	30.0	42.9	239	45.5	50.7	30.0	51.4	486	41.6	47.5	30.0	51.4	229	44.9	50.0	25.7	55.7

IQR = interquartile range | BMI = body mass index | SCB = sugar-containing beverage | bold = significant difference between intervention and control (p < 0.05) | ^a^Sex- and age-specific BMI z-scores according to WHO 2007 criteria [25] | ^b^Weight categories based on the IOTF 2012 criteria [24].

### Intervention effects on adiposity and EBRBs

Table 2 shows that there was no significant intervention effect (c-path) on any of the measures of adiposity. We also found no significant intervention effects on any of the EBRBs outcomes for the whole sample (a-path).

**Table 2:** **Intervention effects on BMI, WC and sum of skinfolds for the whole sample**

	**Intervention effect**	
	**B**	**95% CI**
BMI Z scores (kg/m^2^)^a^	0.03	(−0.02; 0.08)
WC (cm)	0.52	(−0.55; 1.59)
Sum of skinfolds (mm)	0.98	(−1.23; 3.19)
SCB consumption (ml/day)	−56.65	(−177.81; 64.51)
High energy snacks/sweets (portion/day)	0.16	(−0.11; 0.43)
Breakfast consumption (days/week)	0.17	(−0.11; 0.45)
Screen time behaviour (min/day)	15.61	(−9.92; 41.13)
Active transport to school (min/day)	−1.55	(−6.15; 3.06)
Sports participation (min/day)	−1.65	(−8.11; 4.82)

BMI = body mass index | SCB = sugar-containing beverage | WC = waist circumference | Analyses adjusted for age, gender (both not for BMI z-score), baseline values, ethnicity, education | B = regression coefficient | CI = confidence interval | ^a^Sex- and age-specific BMI z-scores according to WHO 2007 criteria [25].

### EBRBs as mediators of intervention effects on adiposity

We found no significant associations (b-path) between the hypothesised EBRB mediators and measures of adiposity (Additional file 1: Appendix 1) and all b-paths consisted of zero values. We also found no significant total mediating effects (a*b). In line with the single-mediator models, the multiple-mediation model did not provide evidence of significant mediation effects by any of the potential mediators (Additional file 1: Appendix 2).

### Moderating effects of gender, ethnicity and education level

Both gender and adolescents’ education level showed significant effect modification. Therefore, analyses were performed for boys and girls separately (Additional file 1: Appendix 3 and 4). In girls, the intervention was effective in reducing SCB consumption (B = −188.2 ml/day; 95% CI = −344.0; −32.3). In boys, we found a significant positive intervention effect on breakfast consumption (B = 0.29 days/week; 95% CI = 0.01; 0.58). In boys, we also found a significant association between total screen time and WC. Screen time did not significantly mediate the intervention effect on WC. Stratified analyses for education level showed an adverse intervention effect (B = 0.09; 95% CI = 0.02; 0.16) on BMI z-scores for adolescents attending the vocational education track (Additional file 1: Appendix 5 and 6). Ethnicity was not a significant effect modifier.

## Discussion

In 2003–2005, the initial DOiT-effectiveness programme showed promising results in a randomised controlled effectiveness trial. Based on the parallel conducted process evaluation, we made adapations to the DOiT programme [21]. The current study tested the effectiveness of the adapted DOiT-implementation programme during natural dissemination. Disappointingly, we found no significant intervention effects on measures of adiposity or the targeted EBRBs. We also found no mediating effects of the EBRBs on adolescents’ adiposity. However, subgroup analyses showed a significant intervention effect on reduced SCB consumption in girls and increased breakfast consumption in boys, and a small but adverse intervention effect on BMI z-scores for adolescents attending the vocational education track.

Notably, our findings deviate from the controlled evaluation of the initial DOiT-effectiveness programme in 2003–2005, where significant effects were found on skinfolds in both boys and girls at 20-months follow-up, WC in boys at 8-months follow-up, a reduction of 250 ml/day of SCB consumption in both boys and girls at 8- and 12-months follow-up, and a reduction in screen time of 25 min/day in boys at 20-months follow-up [18,19]. Regarding mediation effects, analyses of the 2003–2005 data showed that a 140 ml/day decreased SCB consumption led to smaller increases in BMI; that is SCB consumption mediated the intervention effects on BMI (a*b = −0.01; 95% CI = −0.20, −0.001) [20].

There are several possible reasons for the lack of significant effects in the present implementation study: 1) in the present study the intervention was in its dissemination phase and, therefore, less controlled and directed compared to the initial study; 2) methodological issues; 3) the limited room for improvement of the targeted EBRBs; and 4) the different environmental context. The first explanation for the lack of intervention effect is the fact that the programme was evaluated during natural dissemination. We evaluated the natural course of adoption, implementation and maintenance without any guidance from researchers. As a result of the adaptations to the programme, teachers had more flexibility by adding online extra materials with more practical applications for the lessons. Our accompanying process evaluation among teachers indicated that only a few teachers had implemented all lessons and all components of the programme [unpublished observations]. This fidelity problem often occurs during dissemination of school-based prevention programmes. A study by Birnbaum et al. [33] suggested that differences in exposure to multi-component school-based interventions were associated with different outcomes. Since adolescents cannot benefit from a programme they do not receive, this might be one explanation for the lack of effect.

The second possible explanation for the lack of effects is the methodology. Due to the natural dissemination design, we chose to measure outcomes only at baseline and after 20 months during this DOiT-implementation trial, and not at 8- and 12-months follow-up as conducted in the DOiT-effectiveness study. Therefore, we only have insight into 20-months changes and not in the short-term intervention effects. It might be that initial intervention effects, if present, diminished after the intervention period, and were therefore not present in our study at 20-months follow-up.

It might also be that the intervention effects on EBRBs were too small to change adolescents’ adiposity or that a longer follow-up was needed. Furthermore, use of more objective measures, such as observations or accelerometry, may improve the ability to detect effects.

A third possible explanation for the lack of intervention effects might be that there was too little room for improvement for some EBRBs. Whilst behaviours, such as eating breakfast on a daily basis (56%), SCB consumption (1219 ml/day) showed much room for improvement, baseline values for active transport (36 min/day) and sport participation (49 min/day) showed less room for improvement. However, this does not explain the fact that we found no intervention effect on screen time (baseline value of 251 min/day). Both the effectiveness and the implementation DOiT programmes challenged adolescents to set a goal for one out of the five targeted behaviours [14]. It may be easier to improve SCB or breakfast consumption than reduce screen time which is ubiquitous. However, there have been many successful trials that have effectively reduced screen time in youth [34]. Unfortunately, we have no information on what behaviours adolescents chose to improve as part of the DOiT-implementation programme.

A fourth explanation for the lack of intervention effects could be the environmental context. The DOiT-effectiveness trial was conducted in a very different time, characterized by a lower general level of awareness of obesity and its drivers. This is in contrast with the current situation, in which –for example- media attention regarding overweight and obesity and its behavioural risk factors and presumed solutions is present and can be hardly avoided. This could be an additional explanation for the smaller effects observed in the current DOiT-implementation trial as the difference between DOiT and usual care now is likely to be much smaller than in 2003. Although there may not have been a large improvement in behavioural change, avoiding worsening of EBRBs may already be a valuable outcome in the current obesogenic environment. However, since we found the same pattern of changes in behaviours for both the intervention as well as the control group (i.e. no differences for most EBRBs), we cannot attribute these findings to the delivery of the DOiT.

Besides the lack of significant overall effects, the DOiT-implementation programme showed some effects by sub-groups. In girls, we found a significant intervention effect on SCB consumption in both evaluations. Whilst the DOiT-effectiveness evaluation did not succeed in maintaining behavioural change of SCB consumption at 20-months follow-up (girls −88 ml/day; boys −75 ml/day), we found a significant intervention effect on SCB consumption in girls (−188 ml/day) at this time point in the current implementation study. As mentioned previously, we increased the duration of the DOiT-implementation programme from one to two school years. This prolonged duration may have resulted in sustained effects on SCB consumption in girls. In boys, we found a significant positive intervention effect on daily breakfast consumption. The beneficial effect on breakfast consumption is consistent with findings from another study [35] that reported positive intervention effects on breakfast consumption in secondary schools of a 12-week teacher-implemented intervention aimed at improving children’s diet and nutrition knowledge. Considering that breakfast skipping is associated with risk for overweight [36], the intervention effect on daily breakfast consumption is promising. The observed gender differences in intervention effects support earlier findings that girls may benefit from overweight and obesity prevention interventions in different ways than boys [37,38].

Furthermore, the study showed a small but adverse intervention effect on the BMI z-scores for adolescents attending the vocational education track. As mentioned previously, we developed two different versions of the programme, tailored to the subtracks of the prevocational education system in the Netherlands [16]. Our accompanying process evaluation among teachers indicated that the programme was found to be too complex for the vocational student’s education level [unpublished observations]. The observed education level differences in intervention effects support earlier findings that middle and high class children, who are more often attending higher education, benefit more from overweight prevention interventions [39]. Therefore, future overweight and obesity prevention interventions might want to consider developing gender-specific and education level tailored health promotion programmes.

This study has both strengths and limitations. To our knowledge, this is the first study exploring the mediators of school-based intervention effects on measures of adiposity during natural dissemination. Other strengths include the use of objective measures of adiposity according to a standardised measurement protocol, the cluster controlled study design, the large study sample and sub-group analyses.

When interpreting the results, several limitations need to be taken into account. As discussed above, the programme was evaluated during natural dissemination. Thus, the research team could not be blinded to group assignment and the schools were not randomised.

As we evaluated the natural implementation process, it is also possible that adoption bias could have emerged. Since DOiT is an innovative programme, schools that adopted and implemented DOiT may not have been representative of all schools of prevocational education in the Netherlands. It might be that the way schools self-selected themselves may have resulted in participation of schools who were more engaged in other health-promotion programmes than a random sample of schools. Unfortunately, we have no systematic data collected about engagement in other programmes.

Overall the DOiT-implementation programme showed no intervention effects. The subgroup analyses were exploratory. Moreover, the number of subgroup analyses increased the risk of Type I error and therefore our findings need replication in larger samples.

Another limitation is the fact that we used self-report to assess EBRBs. This might have led to social desirability and recall bias. Although the questionnaire showed acceptable reliability and validity [27], the questionnaire targeted only traditional screen devices (i.e., time spent on computer use and television viewing). Adolescents reported to the research team that they found the questions assessing computer and screen time difficult to answer. They told the research team that they often watched television, used the computer and used their mobile phone at the same time. Therefore, we need to consider that the questionnaire does not adequately assess total screen time behaviour.

One more limitation that may have influenced our results is that adolescents who refused to take off their outer clothes (n = 171) at one or two time points were more often overweight (30% vs. 21%) and more often from a Non-Western ethnic group (39% vs. 25%) compared to adolescents who were measured in underwear at both time points. However, there was no significant difference between the number of adolescents who refused to take off their outer clothes between the intervention and control groups. We adjusted body weight for standardised clothing mass values to minimise the influence on our findings.

Finally, we decided to conduct complete cases analyses instead of intention to treat analyses. The latter assumes non-change between baseline and follow-up for non-responders at follow-up. However, because the present study was conducted among adolescents –i.e. an age group in whom natural changes in EBRBs, height and weight because of growth and development are present- assuming no change between baseline and follow-up is not advisable.

## Conclusion

This study underlines the difficulty of translating intervention effectiveness in controlled settings to real world contexts. Under natural conditions, the adapted DOiT-implementation programme was not successful in changing adolescents’ adiposity and no mediating effects were observed*.* However, the programme resulted in beneficial effects on consumption of SCB in girls and breakfast consumption in boys. Based on the results of this study, implementation of the DOiT programme in its present form and with its current implementation strategy should not be endorsed in order to change adolescents’ adiposity. To further improve programme effectiveness, adaptations to the implementation strategy are needed in order to promote implementation as intended by the teachers. Additional programme adaptations by sub-group (e.g., gender, education level) may also be needed. Further, more research is needed to explore what minimal dose of intervention delivery is needed for programme effectiveness. Future studies should continue to evaluate evidence-based programmes during natural dissemination to better understand if and how effectiveness is retained when disseminating evidence-based approaches into practice.

## Additional file

Additional file 1:
**Appendix 1.** Intervention and mediating effects on BMI, WC and sum of skinfolds for the whole sample. **Appendix 2.** Multiple mediator model for mediating effects on BMI, WC and sum of skinfolds. **Appendix 3.** Intervention and mediating effects on BMI, WC and sum of skinfolds for girls. **Appendix 4.** Intervention and mediating effects on BMI, WC and sum of skinfolds for boys. **Appendix 5.** Intervention and mediating effects on BMI, WC and sum of skinfolds for adolescents following the vocational education track. **Appendix 6.** Intervention and mediating effects on BMI, WC and sum of skinfolds for adolescents following the theoretical education track.

## Abbreviations

DOiT: Dutch obesity intervention in teenagers
EBRBs: Energy balance-related behaviours
WC: Waist circumference
SCB: Sugar-containing beverage
BMI: Body mass index
