# Supplementary material for: The Dutch Obesity Intervention in Teenagers (DOiT) cluster controlled implementation trial: intervention effects and mediators and moderators of adiposity and energy balance-related behaviours
Source: Int J Behav Nutr Phys Act. 2014 Dec 24;11:158. doi: 10.1186/s12966-014-0158-0 (PMC4304621; doi:10.1186/s12966-014-0158-0)
Supplement: Additional file 1: — Appendix 1. Intervention and mediating effects on BMI, WC and sum of skinfolds for the whole sample. Appendix 2. Multiple mediator model for mediating effects on BMI, WC and sum of skinfolds. Appendix 3. Intervention and mediating effects on BMI, WC and sum of skinfolds for girls. Appendix 4. Intervention and mediating effects on BMI, WC and sum of skinfolds for boys. Appendix 5. Intervention and mediating effects on BMI, WC and sum of skinfolds for adolescents following the vocational education track. Appendix 6. Intervention and mediating effects on BMI, WC and sum of skinfolds for adolescents following the theoretical education track. [file 12966_2014_158_MOESM1_ESM.docx]

**Appendix 1.** **Intervention and mediating effects on BMI, WC and sum of skinfolds for the whole sample**

|  | Direct (unmediated)  intervention effect  (path c’) | | Intervention Effect on Mediator  (path a) | | Mediator effect on outcome  (path b) | | Mediated effect  (a*b) | |
| --- | --- | --- | --- | --- | --- | --- | --- | --- |
|  | B | 95% CI | B | 95% CI | B | 95% CI | B | 95% CI |
| **BMI Z scores (kg/m^2^)**^a^ | 0.03 | (-0.02 ; 0.08) |  |  |  |  |  |  |
| SCB consumption (ml/day) | 0.02 | (-0.03 ; 0.08) | -56.65 | (-177.81 ; 64.51) | -3.0E-05 | (-6.9E-05 ; 9.2E-06) | 1.7E-03 | (-2.6E-03 ; 6.0E-03) |
| High energy snacks/sweets (portion/day) | 0.03 | (-0.03 ; 0.09) | 0.16 | (-0.11 ; 0.43) | -7.7E-03 | (-2.3E-02 ; 8.1E-03) | -1.2E-03 | (-4.4E-03 ; 2.0E-03) |
| Breakfast consumption (days/week) | 0.02 | (-0.03 ; 0.08) | 0.17 | (-0.11 ; 0.45) | 8.3E-03 | (-5.5E-03 ; 2.2E-02) | 1.4E-03 | (-1.9E-03 ; 4.7E-03) |
| Screen time behaviour (min/day) | 0.03 | (-0.02 ; 0.09) | 15.61 | (-9.92 ; 41.13) | -6.0E-05 | (-2.8E-04 ; 1.6E-04) | -9.4E-04 | (-4.6E-03 ; 2.8E-03) |
| Active transport to school (min/day) | 0.02 | (-0.03 ; 0.08) | -1.55 | (-6.15 ; 3.06) | -3.9E-04 | (-1.7E-03 ; 8.8E-04) | 6.0E-04 | (-2.1E-03 ; 3.3E-03) |
| Sports participation (min/day) | 0.02 | (-0.04 ; 0.08) | -1.65 | (-8.11 ; 4.82) | -3.9E-04 | (-9.8E-04 ; 2.0E-04) | 6.4E-04 | (-2.1E-03 ; 3.3E-03) |
| **WC (cm)** | 0.52 | (-0.55 ; 1.59) |  |  |  |  |  |  |
| SCB consumption (ml/day) | 0.55 | (-0.57 ; 1.67) | -56.65 | (-177.81 ; 64.51) | -7.0E-05 | (-4.4E-04 ; 3.0E-04) | 4.0E-03 | (-1.9E-02 ; 2.7E-02) |
| High energy snacks/sweets (portion/day) | 0.41 | (-0.69 ; 1.51) | 0.16 | (-0.11 ; 0.43) | 3.6E-02 | (-1.3E-01 ; 2.0E-01) | 5.7E-03 | (-2.2E-02 ; 3.3E-02) |
| Breakfast consumption (days/week) | 0.50 | (-0.59 ; 1.58) | 0.17 | (-0.11 ; 0.45) | 4.8E-02 | (-9.5E-02 ; 1.9E-01) | 8.0E-03 | (-2.0E-02 ; 3.6E-02) |
| Screen time behaviour (min/day) | 0.48 | (-0.53 ; 1.50) | 15.61 | (-9.92 ; 41.13) | 1.5E-03 | (-7.3E-04 ; 3.8E-03) | 2.4E-02 | (-2.9E-02 ; 7.7E-02) |
| Active transport to school (min/day) | 0.41 | (-0.68 ; 1.49) | -1.55 | (-6.15 ; 3.06) | -6.6E-03 | (-2.0E-02 ; 6.8E-03) | 1.0E-02 | (-2.6E-02 ; 4.7E-02) |
| Sports participation (min/day) | 0.45 | (-0.60 ; 1.50) | -1.65 | (-8.11 ; 4.82) | -4.0E-03 | (-1.0E-02 ; 2.3E-03) | 6.6E-03 | (-2.1E-02 ; 3.5E-02) |
| **Sum of skinfolds (mm)** | 0.98 | (-1.23 ; 3.19) |  |  |  |  |  |  |
| SCB consumption (ml/day) | 0.70 | (-1.65 ; 3.04) | -56.65 | (-177.81 ; 64.51) | -4.1E-04 | (-1.6E-03 ; 7.5E-04) | 2.3E-02 | (-5.9E-02 ; 1.1E-01) |
| High energy snacks/sweets (portion/day) | 0.96 | (-1.48 ; 3.40) | 0.16 | (-0.11 ; 0.43) | -2.3E-01 | (-7.5E-01 ; 2.9E-01) | 5.2E-02 | (-1.4E-01 ; 6.6E-02) |
| Breakfast consumption (days/week) | 0.84 | (-1.40 ; 3.09) | 0.17 | (-0.11 ; 0.45) | 4.1E-01 | (-3.4E-02 ; 8.6E-01) | 7.0E-02 | (-6.9E-02 ; 2.1E-01) |
| Screen time behaviour (min/day) | 0.94 | (-1.34 ; 3.22) | 15.61 | (-9.92 ; 41.13) | 3.2E-03 | (-3.9E-03 ; 1.0E-02) | 5.0E-02 | (-8.8E-02 ; 1.9E-01) |
| Active transport to school (min/day) | 0.82 | (-1.39 ; 3.03) | -1.55 | (-6.15 ; 3.06) | -1.2E-02 | (-5.4E-02 ; 3.0E-02) | 1.8E-02 | (-6.6E-02 ; 1.0E-01) |
| Sports participation (min/day) | 0.25 | (-2.60 ; 3.10) | -1.65 | (-8.11 ; 4.82) | -1.7E-02 | (-3.6E-02 ; 2.3E-03) | 2.8E-02 | (-8.7E-02 ; 1.4E-01) |

BMI = body mass index | SCB=sugar-containing beverage | WC = waist circumference | Analyses adjusted for age, gender (both not for BMI z-score), baseline values, ethnicity, education | bold = significant p<0.05 | B = regression coefficient | CI = confidence interval |^a^ Sex- and age-specific BMI z-scores according to WHO 2007 criteria [25]

**Appendix 2 Multiple mediator model for mediating effects on BMI, WC and sum of skinfolds**

|  | Intervention Effect on Mediator  (path a) | | Mediator effect on outcome  (path b) | | Mediated effect  (a*b) | |
| --- | --- | --- | --- | --- | --- | --- |
|  | B | 95% CI | B | 95% CI | B | 95% CI |
| **BMI Z scores (kg/m^2^)^a^** |  |  |  |  |  |  |
| SCB consumption (ml/day) | -56.65 | (-177.81 ; 64.51) | -2.0E-05 | (-5.9E-05 ; 1.9E-05) | 1.1E-03 | (-2.2E-03 ; 4.4E-03) |
| High energy snacks/sweets (portion/day) | 0.16 | (-0.11 ; 0.43) | -8.3E-03 | (-3.0E-02 ; 1.4E-02) | -1.3E-03 | (-5.4E-03 ; 2.8E-03) |
| Breakfast consumption (days/week) | 0.17 | (-0.11 ; 0.45) | **1.8E-04** | **(1.8E-04 ; 1.8E-04)** | 3.0E-05 | (-1.9E-02 ; 1.9E-02) |
| Screen time behaviour (min/day) | 15.61 | (-9.92 ; 41.13) | 3.0E-05 | (-2.6E-04 ; 3.2E-04) | 4.7E-04 | (-4.2E-03 ; 5.1E-03) |
| Active transport to school (min/day) | -1.55 | (-6.15 ; 3.06) | -4.9E-04 | (-2.3E-03 ; 1.3E-03) | 7.6E-04 | (-2.8E-03 ; 4.3E-03) |
| Sports participation (min/day) | -1.65 | (-8.11 ; 4.82) | -4.7E-04 | (-1.2E-03 ; 2.7E-04) | 7.7E-04 | (-2.5E-03 ; 4.1E-03) |
| **WC (cm)** |  |  |  |  |  |  |
| SCB consumption (ml/day) | -56.65 | (-177.81 ; 64.51) | -4.0E-05 | (-5.1E-04 ; 4.3E-04) | 2.3E-03 | (-2.5E-02 ; 2.9E-02) |
| High energy snacks/sweets (portion/day) | 0.16 | (-0.11 ; 0.43) | -4.2E-03 | (-2.4E-01 ; 2.3E-01) | -6.5E-04 | (-3.7E-02 ; 3.6E-02) |
| Breakfast consumption (days/week) | 0.17 | (-0.11 ; 0.45) | 4.4E-02 | (-1.5E-01 ; 2.4E-01) | 7.3E-03 | (-1.1E-02 ; 2.6E-02) |
| Screen time behaviour (min/day) | 15.61 | (-9.92 ; 41.13) | 2.4E-03 | (-7.1E-04 ; 5.4E-03) | 3.7E-02 | (-4.0E-02 ; 1.1E-01) |
| Active transport to school (min/day) | -1.55 | (-6.15 ; 3.06) | -1.7E-03 | (-2.0E-02 ; 1.7E-02) | 2.6E-03 | (-2.7E-02 ; 3.2E-02) |
| Sports participation (min/day) | -1.65 | (-8.11 ; 4.82) | -1.8E-03 | (-9.7E-03 ; 6.0E-03) | 3.0E-03 | (-1.4E-02 ; 2.0E-02) |
| **Sum of skinfolds (mm)** |  |  |  |  |  |  |
| SCB consumption (ml/day) | -56.65 | (-177.81 ; 64.51) | -4.6E-04 | (-2.0E-03 ; 1.1E-03) | 2.6E-02 | (-7.8E-02 ; 1.3E-01) |
| High energy snacks/sweets (portion/day) | 0.16 | (-0.11 ; 0.43) | -1.7E-01 | (-9.4E-01 ; 5.9E-01) | -2.7E-02 | (-1.6E-01 ; 1.0E-01) |
| Breakfast consumption (days/week) | 0.17 | (-0.11 ; 0.45) | 8.2E-04 | (-6.4E-01 ; 6.4E-01) | 1.4E-04 | (-1.9E-02 ; 1.9E-02) |
| Screen time behaviour (min/day) | 15.61 | (-9.92 ; 41.13) | -1.8E-03 | (-1.2E-02 ; 8.3E-03) | -2.7E-02 | (-1.9E-01 ; 1.4E-01) |
| Active transport to school (min/day) | -1.55 | (-6.15 ; 3.06) | -1.5E-02 | (-7.6E-02 ; 4.5E-02) | 2.4E-02 | (-9.4E-02 ; 1.4E-01) |
| Sports participation (min/day) | -1.65 | (-8.11 ; 4.82) | **-2.7E-02** | **(-5.3E-02 ; -1.9E-03)** | 4.5E-02 | (-1.4E-01 ; 2.3E-01) |

**Appendix 3.** **Intervention and mediating effects on BMI, WC and sum of skinfolds for girls**

|  | Direct (unmediated)  intervention effect  (path c’) | | Intervention Effect on Mediator  (path a) | | Mediator effect on outcome  (path b) | | Mediated effect  (a*b) | |
| --- | --- | --- | --- | --- | --- | --- | --- | --- |
|  | B | 95% CI | B | 95% CI | B | 95% CI | B | 95% CI |
| **BMI Z scores (kg/m^2^)^a^** | 0.04 | (-0.03 ; 0.11) |  |  |  |  |  |  |
| SCB consumption (ml/day) | 0.05 | (-0.03 ; 0.13) | **-188.17** | **(-344.03 ; -32.31)** | -1.0E-05 | (-6.9E-05 ; 4.9E-05) | 1.9E-03 | (-9.3E-03 ; 1.3E-02) |
| High energy snacks/sweets (portion/day) | 0.04 | (-0.03 ; 0.12) | 0.07 | (-0.27 ; 0.42) | -9.6E-03 | (-3.0E-02 ; 1.1E-02) | -6.9E-04 | (-4.3E-03 ; 2.9E-03) |
| Breakfast consumption (days/week) | 0.03 | (-0.04 ; 0.11) | 0.04 | (-0.25 ; 0.32) | 1.6E-02 | (-2.6E-03 ; 3.4E-02) | 5.8E-04 | (-3.9E-03 ; 5.1E-03) |
| Screen time behaviour (min/day) | 0.05 | (-0.03 ; 0.12) | 10.94 | (-20.25 ; 42.13) | -1.4E-04 | (-4.3E-04 ; 1.5E-04) | -1.5E-03 | (-7.0E-03 ; 3.9E-03) |
| Active transport to school (min/day) | 0.04 | (-0.03 ; 0.12) | -0.23 | (-5.26 ; 4.81) | -4.3E-04 | (-2.2E-03 ; 1.4E-03) | 9.8E-05 | (-2.1E-03 ; 2.3E-03) |
| Sports participation (min/day) | 0.03 | (-0.05 ; 0.10) | -4.33 | (-15.69 ; 7.02) | -1.1E-04 | (-9.9E-04 ; 7.7E-04) | 4.8E-04 | (-3.5E-03 ; 4.5E-03) |
| **WC (cm)** | 0.61 | (-1.07 ; 2.30) |  |  |  |  |  |  |
| SCB consumption (ml/day) | 0.91 | (-0.89 ; 2.72) | **-188.17** | **(-344.03 ; -32.31)** | -7.5E-05 | (-6.0E-04 ; 4.5E-04) | 1.4E-02 | (-8.6E-02 ; 1.1E-01) |
| High energy snacks/sweets (portion/day) | 0.51 | (-1.19 ; 2.20) | 0.07 | (-0.27 ; 0.42) | 4.9E-03 | (-2.1E-01 ; 2.2E-01) | 3.5E-04 | (-1.6E-02 ; 1.6E-02) |
| Breakfast consumption (days/week) | 0.60 | (-1.11 ; 2.31) | 0.04 | (-0.25 ; 0.32) | 1.1E-02 | (-1.8E-01 ; 2.0E-01) | 4.2E-04 | (-7.4E-03 ; 8.2E-03) |
| Screen time behaviour (min/day) | 0.59 | (-0.99 ; 2.18) | 10.94 | (-20.25 ; 42.13) | 8.0E-05 | (-3.2E-03 ; 3.4E-03) | 8.7E-04 | (-3.6E-02 ; 3.7E-02) |
| Active transport to school (min/day) | 0.58 | (-1.06 ; 2.21) | -0.23 | (-5.26 ; 4.81) | -1.1E-02 | (-3.0E-02 ; 7.4E-03) | 2.6E-03 | (-5.4E-02 ; 6.0E-02) |
| Sports participation (min/day) | 0.51 | (-1.17 ; 2.18) | -4.33 | (-15.69 ; 7.02) | -7.2E-03 | (-1.7E-02 ; 2.5E-03) | 3.1E-02 | (-6.1E-02 ; 1.2E-01) |
| **Sum of skinfolds (mm)** | 3.07 | (-0.95 ; 7.09) |  |  |  |  |  |  |
| SCB consumption (ml/day) | 3.62 | (-0.66 ; 7.91) | **-188.17** | **(-344.03 ; -32.31)** | -1.1E-04 | (-1.8E-03 ; 1.6E-03) | 2.1E-02 | (-3.0E-01 ; 3.5E-01) |
| High energy snacks/sweets (portion/day) | 2.97 | (-0.59 ; 6.52) | 0.07 | (-0.27 ; 0.42) | -3.5E-01 | (-1.1E+00 ; 3.8E-01) | 6.8E-02 | (-1.6E-01 ; 1.1E-01) |
| Breakfast consumption (days/week) | 3.05 | (-0.94 ; 7.03) | 0.04 | (-0.25 ; 0.32) | 3.3E-01 | (-2.9E-01 ; 9.6E-01) | 1.2E-02 | (-8.6E-02 ; 1.1E-01) |
| Screen time behaviour (min/day) | 2.97 | (-0.47 ; 6.41) | 10.94 | (-20.25 ; 42.13) | 3.5E-04 | (-1.0E-02 ; 1.1E-02) | 3.8E-03 | (-1.1E-01 ; 1.2E-01) |
| Active transport to school (min/day) | 2.88 | (-0.35 ; 6.10) | -0.23 | (-5.26 ; 4.81) | -1.1E-02 | (-3.0E-02 ; 7.4E-03) | 2.6E-03 | (-5.4E-02 ; 6.0E-02) |
| Sports participation (min/day) | 2.61 | (-1.65 ; 6.87) | -4.33 | (-15.69 ; 7.02) | -1.5E-02 | (-4.6E-02 ; 1.5E-02) | 6.5E-02 | (-1.5E-01 ; 2.8E-01) |

BMI = body mass index | SCB=sugar-containing beverage | WC = waist circumference | Analyses adjusted for age (not for BMI z-score), baseline values, ethnicity, education | bold = significant p<0.05 | B = regression coefficient | CI = confidence interval |^a^ Sex- and age-specific BMI z-scores according to WHO 2007 criteria [25]

**Appendix 4.** **Intervention and mediating effects on BMI, WC and sum of skinfolds for boys**

|  | Direct (unmediated)  intervention effect  (path c’) | | Intervention Effect on Mediator  (path a) | | Mediator effect on outcome  (path b) | | Mediated effect  (a*b) | |
| --- | --- | --- | --- | --- | --- | --- | --- | --- |
|  | B | 95% CI | B | 95% CI | B | 95% CI | B | 95% CI |
| **BMI Z scores (kg/m^2^)^a^** | 0.02 | (-0.06 ; 0.10) |  |  |  |  |  |  |
| SCB consumption (ml/day) | 0.01 | (-0.07 ; 0.10) | 84.78 | (-63.67 ; 233.23) | -2.0E-05 | (-7.9E-05 ; 3.9E-05) | -1.7E-03 | (-7.5E-03 ; 4.1E-03) |
| High energy snacks/sweets (portion/day) | 0.02 | (-0.07 ; 0.11) | 0.23 | (-0.13 ; 0.58) | -4.7E-03 | (-2.9E-02 ; 1.9E-02) | -1.1E-03 | (-6.8E-03 ; 4.7E-03) |
| Breakfast consumption (days/week) | 0.02 | (-0.06 ; 0.09) | **0.29** | **(0.01 ; 0.58)** | 1.5E-03 | (-1.9E-02 ; 2.2E-02) | 4.4E-04 | (-5.7E-03 ; 6.5E-03) |
| Screen time behaviour (min/day) | 0.02 | (-0.06 ; 0.10) | 21.28 | (-5.24 ; 47.81) | 1.2E-04 | (-1.7E-04 ; 4.1E-04) | 2.6E-03 | (-4.5E-03 ; 9.6E-03) |
| Active transport to school (min/day) | 0.01 | (-0.07 ; 0.09) | -2.14 | (-7.55 ; 3.27) | -1.8E-04 | (-2.0E-03 ; 1.6E-03) | 3.9E-04 | (-3.6E-03 ; 4.3E-03) |
| Sports participation (min/day) | 0.02 | (-0.06 ; 0.10) | -1.90 | (-11.13 ; 7.33) | -3.7E-04 | (-1.2E-03 ; 4.3E-04) | 7.0E-04 | (-3.0E-03 ; 4.4E-03) |
| **WC (cm)** | 0.54 | (-0.25 ; 1.33) |  |  |  |  |  |  |
| SCB consumption (ml/day) | 0.33 | (-0.57 ; 1.23) | 84.78 | (-63.67 ; 233.23) | -5.0E-05 | (-5.6E-04 ; 4.6E-04) | -4.2E-03 | (-4.8E-02 ; 4.0E-02) |
| High energy snacks/sweets (portion/day) | 0.53 | (-0.34 ; 1.39) | 0.23 | (-0.13 ; 0.58) | -1.8E-03 | (-2.4E-01 ; 2.3E-01) | -4.1E-04 | (-5.4E-02 ; 5.4E-02) |
| Breakfast consumption (days/week) | 0.48 | (-0.31 ; 1.28) | **0.29** | **(0.01 ; 0.58)** | 6.6E-02 | (-1.4E-01 ; 2.7E-01) | 1.9E-02 | (-4.4E-02 ; 8.3E-02) |
| Screen time behaviour (min/day) | 0.50 | (-0.29 ; 1.30) | 21.28 | (-5.24 ; 47.81) | **3.0E-03** | **(6.1E-05 ; 6.0E-03)** | 6.5E-02 | (-3.8E-02 ; 1.7E-01) |
| Active transport to school (min/day) | 0.46 | (-0.35 ; 1.27) | -2.14 | (-7.55 ; 3.27) | -1.3E-03 | (-1.9E-02 ; 1.7E-02) | 2.8E-03 | (-3.6E-02 ; 4.2E-02) |
| Sports participation (min/day) | 0.55 | (-0.29 ; 1.38) | -1.90 | (-11.13 ; 7.33) | -3.2E-03 | (-1.1E-02 ; 5.0E-03) | 6.1E-03 | (-2.7E-02 ; 4.0E-02) |
| **Sum of skinfolds (mm)** | -0.91 | (-3.63 ; 1.81) |  |  |  |  |  |  |
| SCB consumption (ml/day) | -1.83 | (-4.45 ; 0.78) | 84.78 | (-63.67 ; 233.23) | -3.2E-04 | (-1.8E-03 ; 1.2E-03) | -2.7E-02 | (-1.6E-01 ; 1.1E-01) |
| High energy snacks/sweets (portion/day) | -1.45 | (-4.09 ; 1.20) | 0.23 | (-0.13 ; 0.58) | 1.5E-01 | (-5.8E-01 ; 8.8E-01) | 8.9E-02 | (-1.4E-01 ; 2.1E-01) |
| Breakfast consumption (days/week) | -1.26 | (-4.06 ; 1.55) | **0.29** | **(0.01 ; 0.58)** | 5.5E-01 | (-7.2E-02 ; 1.2E+00) | 1.6E-01 | (-7.8E-02 ; 4.0E-01) |
| Screen time behaviour (min/day) | -1.45 | (-4.69 ; 1.80) | 21.28 | (-5.24 ; 47.81) | 6.9E-03 | (-2.2E-03 ; 1.6E-02) | 1.5E-01 | (-1.2E-01 ; 4.1E-01) |
| Active transport to school (min/day) | -1.01 | (-3.46 ; 1.44) | -2.14 | (-7.55 ; 3.27) | 6.2E-03 | (-4.9E-02 ; 6.1E-02) | -1.3E-02 | (-1.4E-01 ; 1.1E-01) |
| Sports participation (min/day) | -1.99 | (-5.27 ; 1.30) | -1.90 | (-11.13 ; 7.33) | -2.4E-02 | (-4.8E-02 ; 8.8E-04) | 4.5E-02 | (-1.8E-01 ; 2.7E-01) |

BMI = body mass index | SCB=sugar-containing beverage | WC = waist circumference | Analyses adjusted for age (not for BMI z-score), baseline values, ethnicity, education | bold = significant p<0.05 | B = regression coefficient | CI = confidence interval |^a^ Sex- and age-specific BMI z-scores according to WHO 2007 criteria [25]

**Appendix 5.** **Intervention and mediating effects on BMI, WC and sum of skinfolds for adolescents following the vocational education track**

|  | Direct (unmediated)  intervention effect  (path c’) | | Intervention Effect on Mediator  (path a) | | Mediator effect on outcome  (path b) | | Mediated effect  (a*b) | |
| --- | --- | --- | --- | --- | --- | --- | --- | --- |
|  | B | 95% CI | B | 95% CI | B | 95% CI | B | 95% CI |
| **BMI Z scores (kg/m^2^)^a^** | **0.09** | **(0.02 ; 0.16)** |  |  |  |  |  |  |
| SCB consumption (ml/day) | 0.07 | (-0.01 ; 0.16) | -108.88 | (-278.44 ; 60.68) | **4.0E-05** | **(8.0E-07 ; 7.9E-05)** | -4.4E-03 | (-1.2E-02 ; 3.7E-03) |
| High energy snacks/sweets (portion/day) | **0.10** | **(0.01 ; 0.18)** | 0.02 | (-0.34 ; 0.37) | -3.1E-03 | (-2.7E-02 ; 2.1E-02) | -5.5E-05 | (-1.2E-03 ; 1.1E-03) |
| Breakfast consumption (days/week) | **0.08** | **(0.01 ; 0.16)** | 0.23 | (-0.18 ; 0.65) | 1.6E-02 | (-2.1E-03 ; 3.3E-02) | 3.6E-03 | (-4.0E-03 ; 1.1E-02) |
| Screen time behaviour (min/day) | **0.09** | **(0.01 ; 0.17)** | 11.22 | (-13.15 ; 35.59) | 1.1E-04 | (-1.8E-04 ; 4.0E-04) | 1.2E-03 | (-3.0E-03 ; 5.5E-03) |
| Active transport to school (min/day) | **0.08** | **(0.01 ; 0.16)** | -2.68 | (-8.27 ; 2.91) | -8.6E-04 | (-2.5E-03 ; 8.3E-04) | 2.3E-03 | (-4.3E-03 ; 8.9E-03) |
| Sports participation (min/day) | **0.08** | **(0.00 ; 0.16)** | 5.13 | (-1.88 ; 12.13) | **-1.1E-03** | **(-1.9E-03 ; -3.0E-04)** | -5.7E-03 | (-1.5E-02 ; 3.2E-03) |
| **WC (cm)** | 1.17 | (-0.17 ; 2.51) |  |  |  |  |  |  |
| SCB consumption (ml/day) | 1.14 | (-0.34 ; 2.62) | -108.88 | (-278.44 ; 60.68) | -4.2E-04 | (-9.5E-04 ; 1.1E-04) | 4.6E-02 | (-5.3E-05 ; 9.2E-02) |
| High energy snacks/sweets (portion/day) | 1.12 | (-0.29 ; 2.52) | 0.02 | (-0.34 ; 0.37) | -1.7E-03 | (-2.6E-01 ; 2.6E-01) | -3.1E-05 | (-4.8E-03 ; 4.7E-03) |
| Breakfast consumption (days/week) | 1.07 | (-0.31 ; 2.45) | 0.23 | (-0.18 ; 0.65) | 1.0E-01 | (-9.4E-02 ; 2.9E-01) | 2.3E-02 | (-3.8E-02 ; 8.5E-02) |
| Screen time behaviour (min/day) | 1.12 | (-0.22 ; 2.45) | 11.22 | (-13.15 ; 35.59) | 2.9E-03 | (-2.5E-04 ; 6.1E-03) | 3.3E-02 | (-4.7E-02 ; 1.1E-01) |
| Active transport to school (min/day) | 0.99 | (-0.33 ; 2.31) | -2.68 | (-8.27 ; 2.91) | -1.5E-02 | (-3.4E-02 ; 3.7E-03) | 4.1E-02 | (-5.8E-02 ; 1.4E-01) |
| Sports participation (min/day) | 1.06 | (-0.27 ; 2.40) | 5.13 | (-1.88 ; 12.13) | -9.0E-03 | (-1.8E-02 ; 4.5E-04) | -4.6E-02 | (-1.3E-01 ; 3.3E-02) |
| **Sum of skinfolds (mm)** | 0.89 | (-2.28 ; 4.06) |  |  |  |  |  |  |
| SCB consumption (ml/day) | -0.03 | (-2.87 ; 2.81) | -108.88 | (-278.44 ; 60.68) | -1.1E-03 | (-1.7E-02 ; 1.5E-02) | 1.2E-01 | (-1.7E+00 ; 1.9E+00) |
| High energy snacks/sweets (portion/day) | 0.74 | (-2.84 ; 4.31) | 0.02 | (-0.34 ; 0.37) | -1.3E-01 | (-9.5E-01 ; 7.0E-01) | 2.4E-02 | (-5.0E-02 ; 4.5E-02) |
| Breakfast consumption (days/week) | 0.55 | (-2.67 ; 3.77) | 0.23 | (-0.18 ; 0.65) | **7.0E-01** | **(1.2E-01 ; 1.3E+00)** | 1.6E-01 | (-1.6E-01 ; 4.9E-01) |
| Screen time behaviour (min/day) | 0.63 | (-2.69 ; 3.95) | 11.22 | (-13.15 ; 35.59) | 7.7E-03 | (-2.0E-03 ; 1.7E-02) | 8.7E-02 | (-1.3E-01 ; 3.0E-01) |
| Active transport to school (min/day) | 0.58 | (-2.53 ; 3.69) | -2.68 | (-8.27 ; 2.91) | -8.8E-03 | (-6.6E-02 ; 4.9E-02) | 2.3E-02 | (-1.4E-01 ; 1.8E-01) |
| Sports participation (min/day) | 0.32 | (-2.90 ; 3.54) | 5.13 | (-1.88 ; 12.13) | **-4.4E-02** | **(-7.2E-02 ; -1.7E-02)** | -2.3E-01 | (-5.7E-01 ; 1.1E-01) |

BMI = body mass index | SCB=sugar-containing beverage | WC = waist circumference | Analyses adjusted for age, gender (both not for BMI z-score), baseline values, ethnicity | bold = significant p<0.05 | B = regression coefficient | CI = confidence interval |^a^ Sex- and age-specific BMI z-scores according to WHO 2007 criteria [25]

**Appendix 6.** **Intervention and mediating effects on BMI, WC and sum of skinfolds for adolescents** **following the** **theoretical education track**

|  | Direct (unmediated)  intervention effect  (path c’) | | Intervention Effect on Mediator  (path a) | | Mediator effect on outcome  (path b) | | Mediated effect  (a*b) | |
| --- | --- | --- | --- | --- | --- | --- | --- | --- |
|  | B | 95% CI | B | 95% CI | B | 95% CI | B | 95% CI |
| **BMI Z scores (kg/m^2^)^a^** | -0.04 | (-0.12 ; 0.03) |  |  |  |  |  |  |
| SCB consumption (ml/day) | -0.03 | (-0.11 ; 0.06) | 3.75 | (-124.95 ; 132.44) | 3.0E-06 | (-5.0E-05 ; 5.6E-05) | 1.1E-05 | (-4.2E-04 ; 4.5E-04) |
| High energy snacks/sweets (portion/day) | -0.05 | (-0.13 ; 0.03) | 0.23 | (-0.17 ; 0.63) | -1.1E-02 | (-3.2E-02 ; 9.5E-03) | -2.6E-03 | (-9.2E-03 ; 4.0E-03) |
| Breakfast consumption (days/week) | -0.05 | (-0.12 ; 0.03) | 0.15 | (-0.11 ; 0.40) | -2.9E-03 | (-2.5E-02 ; 1.9E-02) | -4.3E-04 | (-3.8E-03 ; 2.9E-03) |
| Screen time behaviour (min/day) | -0.03 | (-0.11 ; 0.05) | 9.10 | (-24.69 ; 42.88) | -2.9E-04 | (-6.2E-04 ; 2.7E-05) | -2.7E-03 | (-1.3E-02 ; 7.7E-03) |
| Active transport to school (min/day) | -0.05 | (-0.13 ; 0.03) | -0.70 | (-5.18 ; 3.79) | 3.6E-04 | (-1.6E-03 ; 2.3E-03) | -2.5E-04 | (-2.3E-03 ; 1.8E-03) |
| Sports participation (min/day) | -0.03 | (-0.11 ; 0.05) | -9.31 | (-18.99 ; 0.37) | 3.5E-04 | (-4.8E-04 ; 1.2E-03) | -3.2E-03 | (-1.2E-02 ; 5.2E-03) |
| **WC (cm)** | -0.41 | (-1.53 ; 0.71) |  |  |  |  |  |  |
| SCB consumption (ml/day) | -0.29 | (-1.30 ; 0.73) | 3.75 | (-124.95 ; 132.44) | 3.3E-04 | (-1.8E-04 ; 8.3E-04) | 1.2E-03 | (-4.1E-02 ; 4.4E-02) |
| High energy snacks/sweets (portion/day) | -0.05 | (-1.27 ; 1.17) | 0.23 | (-0.17 ; 0.63) | 5.2E-02 | (-1.5E-01 ; 2.5E-01) | 1.2E-02 | (-3.9E-02 ; 6.3E-02) |
| Breakfast consumption (days/week) | -0.26 | (-1.44 ; 0.92) | 0.15 | (-0.11 ; 0.40) | -3.0E-02 | (-2.5E-01 ; 1.9E-01) | -4.4E-03 | (-3.7E-02 ; 2.8E-02) |
| Screen time behaviour (min/day) | -0.26 | (-1.41 ; 0.88) | 9.10 | (-24.69 ; 42.88) | -9.1E-04 | (-4.1E-03 ; 2.3E-03) | -8.3E-03 | (-5.1E-02 ; 3.4E-02) |
| Active transport to school (min/day) | -0.35 | (-1.54 ; 0.85) | -0.70 | (-5.18 ; 3.79) | 6.2E-03 | (-1.2E-02 ; 2.5E-02) | -4.3E-03 | (-3.5E-02 ; 2.6E-02) |
| Sports participation (min/day) | -0.15 | (-1.31 ; 1.02) | -9.31 | (-18.99 ; 0.37) | 1.4E-03 | (-6.8E-03 ; 9.5E-03) | -1.3E-02 | (-9.0E-02 ; 6.5E-02) |
| **Sum of skinfolds (mm)** | 1.52 | (-1.59 ; 4.62) |  |  |  |  |  |  |
| SCB consumption (ml/day) | 1.94 | (-0.65 ; 4.52) | 3.75 | (-124.95 ; 132.44) | 1.5E-04 | (-1.5E-03 ; 1.8E-03) | 5.6E-04 | (-2.0E-02 ; 2.1E-02) |
| High energy snacks/sweets (portion/day) | 0.65 | (-2.92 ; 4.23) | 0.23 | (-0.17 ; 0.63) | -4.2E-01 | (-1.1E+00 ; 2.3E-01) | 1.1E-01 | (-3.2E-01 ; 1.3E-01) |
| Breakfast consumption (days/week) | 0.80 | (-2.77 ; 4.37) | 0.15 | (-0.11 ; 0.40) | 5.0E-02 | (-6.6E-01 ; 7.6E-01) | 7.3E-03 | (-9.7E-02 ; 1.1E-01) |
| Screen time behaviour (min/day) | 1.47 | (-1.01 ; 3.96) | 9.10 | (-24.69 ; 42.88) | -4.3E-03 | (-1.5E-02 ; 5.9E-03) | -3.9E-02 | (-2.1E-01 ; 1.3E-01) |
| Active transport to school (min/day) | 0.69 | (-2.93 ; 4.32) | -0.70 | (-5.18 ; 3.79) | -1.4E-02 | (-7.5E-02 ; 4.7E-02) | 9.9E-03 | (-6.7E-02 ; 8.6E-02) |
| Sports participation (min/day) | 0.70 | (-2.95 ; 4.36) | -9.31 | (-18.99 ; 0.37) | 1.3E-02 | (-1.3E-02 ; 4.0E-02) | -1.2E-01 | (-4.0E-01 ; 1.6E-01) |

BMI = body mass index | SCB=sugar-containing beverage | WC = waist circumference | Analyses adjusted for age, gender (both not for BMI z-score), baseline values, ethnicity | bold = significant p<0.05 | B = regression coefficient | CI = confidence interval |^a^ Sex- and age-specific BMI z-scores according to WHO 2007 criteria [25]
